# Risk of progression to eosinophilic esophagitis in patients with asymptomatic esophageal eosinophilia: A retrospective pilot study

**DOI:** 10.1002/jgh3.12270

**Published:** 2019-10-11

**Authors:** Fumiaki Ishibashi, Keita Fukushima, Ryoichi Onizuka, Ryu Tanaka

**Affiliations:** ^1^ Shinjuku Tsurukame Clinic Digestive Disease Center Tokyo Japan; ^2^ Koganei Tsurukame Clinic Endoscopic Center Tokyo Japan; ^3^ Department of Gastroenterology and Hepatology Tokyo Medical and Dental University Tokyo Japan

**Keywords:** asymptomatic esophageal eosinophilia, eosinophilic esophagitis, esophageal eosinophilia

## Abstract

**Background and Aim:**

As the number of patients with eosinophilic esophagitis (EoE) has increased worldwide, the likelihood of diagnosing esophageal eosinophilia (EE) in screening endoscopy has also increased. Many of these EE patients do not display any symptoms (i.e. they display asymptomatic EE: aEE), and the risk of aEE patients developing EoE has yet to be demonstrated.

**Methods:**

A total of 62 250 cases were found in the endoscopic registries of two digestive disease centers in the context of gastric cancer screening from April 2016 to August 2018, and these were reviewed.

**Results:**

Thirty‐seven aEE patients (0.059%) were found in the registries, and the histories of endoscopic findings and symptoms were successfully traced for 29 of them. While 11 aEE (37.9%) patients did not show any change in endoscopic findings, 18 (62.1%) exhibited exacerbation. A comparison of the two groups showed both relative youth and diffuse disease distribution to be independent risk factors for progression (*P* = 0.0034 and 0.0078, respectively). Of the 18 aEE patients whose findings showed progression, 6 developed EoE (5 (17.2%) developed proton‐pump inhibitor (PPI)‐responsive EoE, and only 1 (3.4%) developed PPI‐resistant EoE). A comparison of the non‐EoE and EoE groups showed relative youth to be an independent risk factor for progression to EoE (*P* = 0.0146).

**Conclusions:**

While some aEE patients developed symptomatic EE, the existence among them of PPI‐resistant EoE was extremely rare. Younger age and diffuse disease distribution at first detection in endoscopic findings are risk factors for progression to symptomatic EE in aEE patients.

## Graphical abstract

In this study, the authors presented a hypothesis about the natural history of asymptomatic esophageal eosinophilia (aEE). Some aEE patients progressed to EoE. By analyzing the large healthy cohort, younger age and diffuse disease distribution at the first detection of disease might be considered risk factors of the progression to EoE in aEE patients.

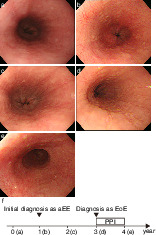

## Introduction

Eosinophilic esophagitis (EoE) was first reported in 1993 and is defined as an inflammatory disease of the esophagus that is characterized by significant infiltration of eosinophils into the esophageal mucosa.^1^, ^2^ The existence of more than 15 eosinophils per high power field (HPF) in esophagus mucosa satisfies the criteria for diagnosing EoE.^3^, ^4^, ^5^ These eosinophils contribute to the development of local inflammation and tissue damage through a Th2 type‐dominant allergic reaction,^6^ and EoE patients exhibit symptoms such as dysphagia, food impaction, chest pain, nausea, and vomiting. The severity of EoE varies among individual patients. Esophageal stricture can occur in patients with long‐term histories of EoE, and this can lead to a worsening in the quality of life.^7^, ^8^ In addition, the existence of patients with esophageal eosinophilia (EE) who do not have a symptomatic history (i.e. who exhibit asymptomatic esophageal eosinophilia [aEE]) has been detected in endoscopic tests for cancer screening.^9^, ^10^, ^11^, ^12^ In Japan, it is particularly common to perform such examinations, even on healthy people, and thus, the likelihood of encountering patients with aEE is higher than in many other countries.^13^, ^14^ Endoscopically screened aEE shares common features with EoE: linear furrowing, concentric rings, and white speckled exudates. The existence of such features indicates the advisability of performing pathological diagnoses on aEE patients.^9^, ^10^, ^11^

The endoscopic features of EoE have come to be commonly recognized by physicians who perform endoscopies, and the prevalence of EoE diagnoses has consequently increased in a number of countries, including in Japan.^15^, ^16^, ^17^ Recent investigations into the epidemiology of EoE have demonstrated a higher prevalence than had previously been predicted.^18^ The current prevalence of EoE is about 1 to 6 per 10 000 persons in Western countries. While this prevalence of aEE may in fact be much higher than that of EoE, there are few studies on the epidemiology of aEE.^19^, ^20^ Furthermore, it has yet to be determined whether aEE can develop into EoE. In this study, we have evaluated the prevalence of aEE in the healthy portion of the original endoscopic registry patients and have investigated the relationship between aEE and EoE.

## Methods

### *Subjects*

Two digestive disease centers for cancer screening, Shinjuku Tsurukame Clinic (located in the central urban area of Tokyo) and Koganei Tsurukame Clinic (located in a suburban city further to the west, within Tokyo Metropolis), were used for this study. A total of 62 250 people underwent routine gastrointestinal endoscopies for cancer screening from April 2016 to August 2018, and their records were subsequently analyzed. In our clinics, the cancer screening program has been conducted every year in order not to miss the diminutive lesions in the stomach. Patients diagnosed with EE were chosen for inclusion in the study. EE was diagnosed when a biopsy sample exhibited significant eosinophil infiltration into the esophageal mucosa (more than 15 cells/HPF). Biopsy samples were taken from at least three distinct regions of the esophagus. Patients exhibiting increased numbers of eosinophils were excluded if they also suffered from gastroesophageal reflux disease (GERD) or other infectious diseases. The medical records of EE patients were reviewed, and information was obtained related to relevant patient characteristics, the status of *Helicobacter pylori* (HP) infection, therapy previously received, and prognoses. When patients with EE showed symptoms (e.g. dysphagia, food impaction, chest pain, nausea, vomiting, etc.), they were diagnosed with EoE and were excluded from our protocol. Finally, when patients diagnosed with aEE had received other endoscopic examinations, either before or after that diagnosis, the results of all their examinations were taken into consideration and analyzed. Patients diagnosed with aEE who had not received other such endoscopic examinations were excluded as subjects for our study. This study protocol was approved by the Ethics Committee of the Shinjuku Tsurukame Clinic (Approval number: 1802).

### *Endoscopic findings*

All endoscopic pictures of aEE patients were subsequently reviewed by three different physicians in a blind examination, and subsequent analyses were performed on findings that two or more reviewers agreed merited further attention. Significant endoscopic findings included the existence of linear furrowing, mucosal edema, and a range of lesions. In this study, we classified lesion range into two types: limited type (just above the esophageal–gastric junction) or diffuse type (middle to lower part of the esophagus). Esophageal ring is known to be one of the efficient findings that predict the degree of fibrosis in lamina propria; however, we did not assess the findings in this study because it was difficult for reviewers to decide whether the esophageal ring existed or not using a limited number of endoscopic pictures. In this study, the status of disease progression was defined as changes in endoscopic findings, namely, extension into diffuse type from limited type of lesion range or appearance of mucosal edema onto linear furrowing.

### *Statistical analyses*

All statistical analyses were performed with SAS 9.4 (University Edition, SAS Institute Inc., Tokyo, Japan). To compare data averages between two independent groups, Student's t‐tests were performed, and results were expressed in terms of mean ± standard error of mean. For comparing two independent non‐numerical datasets, Fisher's exact tests were performed, and *P* values were evaluated. Logistic regression analyses were performed for explanatory variables with significant differences, and *P* values were evaluated. *P* values less than 0.05 were regarded to be statistically significant.

## Results

### *Risk of disease progression among aEE patients as indicated by endoscopic findings*

**Figure 1:**
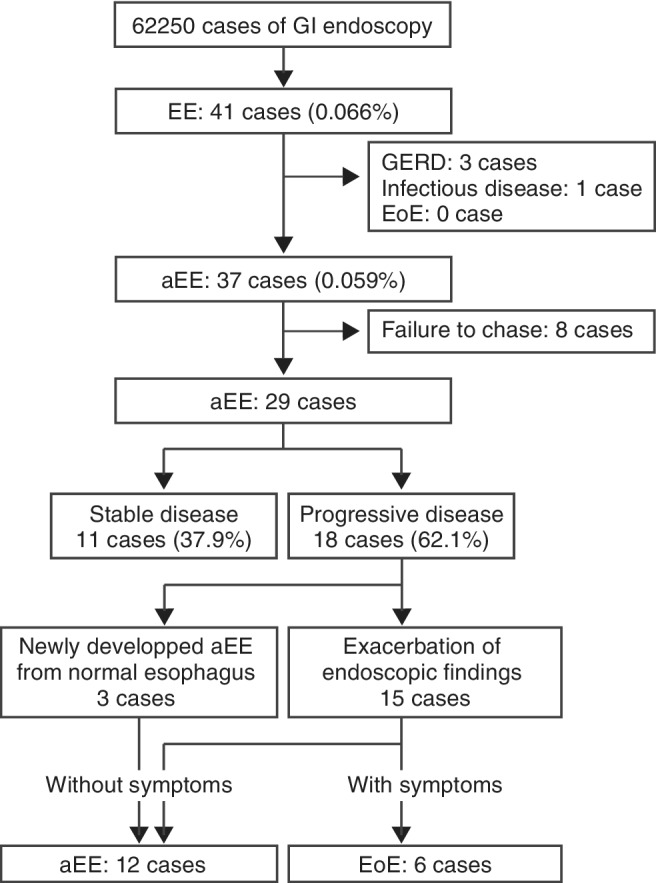
The scheme of inclusion and exclusion criteria of subjects and the strategy for analysis. The 29 cases used in our study were classified into a stable disease group and a progressive disease group. The progressive disease group was subsequently divided into two subgroups, an aEE group and an EoE group. The aEE group consisted of two populations, patients for whom aEE was newly developed from a normal esophagus, without symptoms, and aEE patients whose conditions were exacerbated, without symptoms, during chasing periods. aEE, asymptomatic esophageal eosinophilia; EoE, eosinophilic esophagitis; GERD, gastroesophageal reflux disease.

As a baseline characteristics of our whole cohort, age distribution and gender ratio were shown in Figure S1, Supporting information. A total of 41 cases (0.066%) of EE were found in the database. Of those, three cases of GERD and one case of infectious disease (esophagitis caused by Herpes simplex virus 1) were excluded. There was no EoE patient in our registries at the initial inclusion. The remaining 37 cases (0.059%) of EE were diagnosed as aEE on the basis of a review of medical records. Of the 37, 29 had received two or more endoscopic examinations, and these were selected for analysis (Fig. 1). The mean period of observation was 40.1 ± 4.0 months. Of the 29, 18 cases (62.1%) exhibited disease progression (3 cases of newly developed aEE and 15 cases exacerbated aEE). The remaining 11 cases (37.9%) showed no change in endoscopic findings. Of the 11 cases of the stable disease group, 1 case had findings that slightly improved in 24 months, but the lesion of linear furrowing did not completely disappear. The baseline characteristics of the two groups (*n* = 18 and 11) are shown in Table 1. For the two groups, there was no statistical difference in the period of time for which endoscopic findings were obtained (41.1 *vs* 33.2 months; *P* = 0.31). As in previous studies regarding EoE, here, males were found to be more susceptible to eosinophilic infiltration than females, but there was no statistical difference between the two in terms of proportion within the individual groups (15:3 *vs* 7:4; *P* = 0.1741). Interestingly, relative youth at the time of original lesion detection was found to be a risk factor for disease progression (average ages of 42.8 *vs* 51.8 years; *P* = 0.0034). In addition, the extent of distribution of endoscopic findings (longitudinal length of linear furrowing; diffuse or limited lesion range) differed between the Progressive and Stable groups. Diffuse type was found to be another risk for disease progression (66.7 *vs* 22.2%; *P* = 0.0078). Multivariable regression analysis confirmed the significance of these two factors (relative youth and diffuse type of distribution) with respect to risk of exacerbation (*P* = 0.00728 and 0.00778). We also considered the idea that recovering gastric acid production due to HP eradication therapy increased the risk of the occurrence of GERD, which would exacerbate existing EoE, and we investigated the possible connection between HP eradication therapy and exacerbation, but we found none (22.2 *vs* 27.2%; *P* = 1.000). Furthermore, a history of allergies, including asthma, was found not to be a significant risk for exacerbation of aEE (27.8 *vs* 9.1%; *P* = 0.3623). These results suggest that the respective disease etiologies of aEE and EoE did not correspond perfectly but that there is significant overlap between the two.

**Table 1:** Baseline characteristics and endoscopic findings for progressive disease group and stable disease group

	Progressive disease (*n* = 18)	Stable disease (*n* = 11)	Univariate analysis	Multivariate analysis
Chasing period (months)	41.1	33.2	*P* = 0.3100	
Age	42.8	51.8	*P* = 0.0034	*P* = 0.00728
Gender (male: female)	15:3	7:4	*P* = 0.1741	*P* = 0.9015
Disease distribution (rate of diffuse type [%])	12 (66.7)	2 (22.2)	*P* = 0.0078	*P* = 0.00778
Status of HP infection (rate of receiving HP eradication therapy [%])	4 (22.2)	3 (27.2)	*P* = 1.0000	*P* = 0.1791
History of allergies (%)	5 (27.8)	1 (9.1)	*P* = 0.3623	*P* = 0.5597

HP, *Helicobacter Pylori*.

### *Potential for developing EoE in aEE patients*

**Figure 2:**
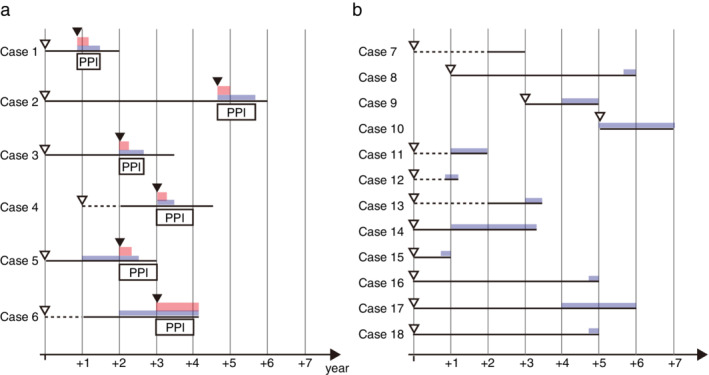
Time courses for individual cases in the progressive disease group. (a) Time course of EoE group (Cases 1–6). All patients received PPI therapy after a diagnosis of EoE. Cases 1–5 immediately responded to PPI and were regarded as PPI‐responsive esophageal eosinophilia. Case 6 showed resistance to PPI therapy, as well as disease progression. (b) Time course for the aEE group (Cases 7–18). Four cases (Case 7 and Cases 11–13) exhibited a limited type of linear furrowing. While 8 cases exhibited a diffuse type of linear furrowing from the time of initial disease diagnosis, and 11 of 12 cases (Cases 8–18) displayed mucosal edema over the course of a number of years of observation, and no cases developed into EoE. (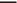), Linear furrowing (diffuse); (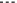), linear furrowing (limited); (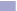), mucosal edema; (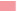), symptom; (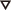), initial diagnosis as EE; (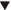), diagnosis as EoE; (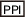), treatment with PPI. aEE, asymptomatic esophageal eosinophilia; EoE, eosinophilic esophagitis; PPI, proton‐pump inhibitor.

**Figure 3:**
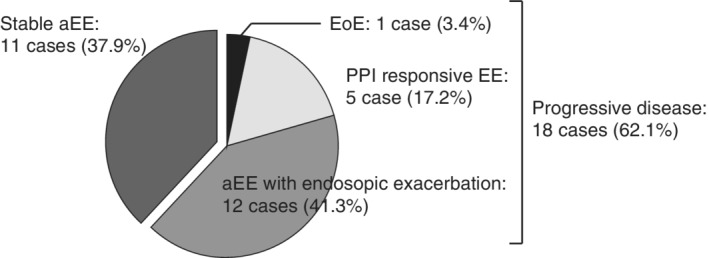
Case details. Eleven cases (37.9%) did not show any change in endoscopic findings. Twelve cases (41.3%) exhibited exacerbation in endoscopic findings but did not show any symptoms. Five cases (17.2%) developed PPI‐responsive esophageal eosinophilia. Only one case (3.4) developed EoE that did not respond to PPI therapy. aEE, asymptomatic esophageal eosinophilia; EoE, eosinophilic esophagitis; PPI, proton‐pump inhibitor.

In order to try to determine the as‐yet‐unknown potential for aEE patients to develop EoE, we analyzed cases that developed EoE during chasing periods. The time courses of disease progression shown in endoscopic findings and in the occurrence of symptoms among the 18 cases in the Progressive disease group are shown in Figure 2. Six cases (Cases 1–6) displayed esophagus‐related symptoms (Case 1 had persistent heartburn; Cases 4 and 5 had chest pain; and Cases 2, 3, and 6 had food impaction) and were newly diagnosed with EoE on the basis of endoscopic examinations and rebiopsies of esophageal mucosa. All six cases exhibited increased collagen deposition in the lamina propria on specimens, which was consistent with disease progression. All six cases received a proton‐pump inhibitor (PPI) (rabeprazole 10 mg a day or esomeprazole 20 mg a day) for 8 weeks. Among them, five cases (Cases 1–5) responded to the PPI, and their symptoms completely disappeared within 6 months to 1 year (EE responsive to PPI: 17.2%). All five cases showed histological improvement, including eosinophil infiltration. One case (Case 6), however, was resistant to PPI and required stronger therapy (prednisolone) and exhibited histological worsening (EoE: 3.4%) (Fig. 3). Notably, as five of six cases exhibited a degree of aEE at the time of their first endoscopic examinations, it is uncertain whether the rate of disease progression depended on the time that may have elapsed after any possible change in esophageal mucosa. In contrast, 12 cases (Cases 7–18) showed disease progression in endoscopic findings but did not exhibit any esophageal symptoms (aEE with endoscopic exacerbation: 41.3%). These 12 cases were not diagnosed with EoE and were continually observed over the years, without any treatment being given. The onset of linear furrowing was detected in only three cases (Cases 8, 9, and 10), but the respective time periods between their initial examinations and the detection of disease progression differed significantly (Case 8 showed a long‐term stable period preceding disease progression, Case 9 showed a short‐term stable period preceding a rapid disease progression of 1 year, and Case 10 showed a stable period of 2 years).

### *Risk of developing EoE in aEE patients*

**Figure 4:**
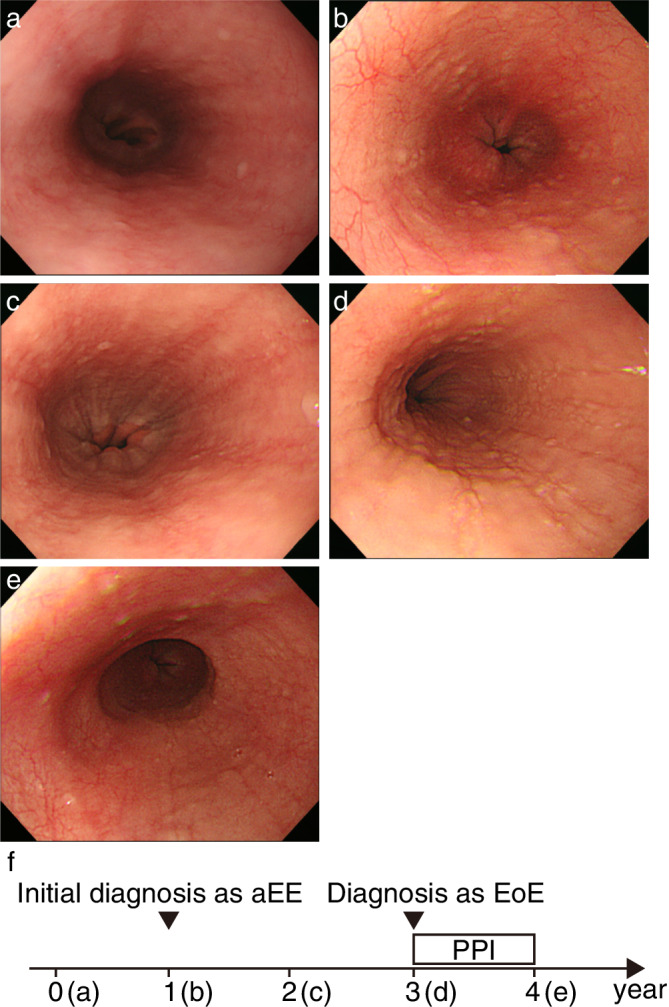
Representative endoscopic pictures during time course of Case 4. (a) Picture taken 1 year before the initial diagnosis as aEE did not exhibit any lesion. (b) Limited type of linear furrowing was found, and biopsy samples taken from linear furrowing confirmed the diagnosis as aEE at the time. (c) Linear furrowing extended to diffuse type 1 year after the initial diagnosis. (d) The patient complained of chest pain. Follow‐up endoscopy exhibited the emergence of mucosal edema onto linear furrowing. The patient was subsequently diagnosed with EoE and received PPI for 1 year. The symptoms rapidly disappeared in 1 month. (e) After treatment with PPI, the endoscopic findings of mucosal edema improved, but linear furrowing remained unchanged. (f) The scheme of follow‐up endoscopy. Words within brackets mean the corresponding figures. aEE, asymptomatic esophageal eosinophilia; EoE, eosinophilic esophagitis; PPI, proton‐pump inhibitor.

To clarify the risk of developing EoE in aEE patients, we classified the Progressive disease group into two subgroups (EoE group [Cases 1–6] and aEE group [Cases 7–18]) and compared baseline characteristics and endoscopic features. While EoE is defined as resistant to PPI therapy in a narrow sense of the definition, we chose to include PPI‐responsive EE cases as well in the EoE grouping in terms of their need for treatment. Baseline characteristics for the two groups are shown in Table 2. There is no difference between them with respect to the chasing period (39.6 *vs* 43.8 months; *P* = 0.733). Periods until endoscopic findings showed that exacerbation was not significantly different (27 *vs* 36 months; *P* = 0.417). As in the comparison between the progressive disease and stable disease groups, the EoE group was significantly younger than the aEE group (average ages of 38.2 *vs* 45.9 years; *P* = 0.0146). Multivariable regression analysis confirmed that a younger age was a significant risk factor for developing EoE in aEE patients (*P* = 0.00643). Unlike in the comparison between the progressive disease and stable disease groups, disease distribution at first detection was not a significant risk factor for progression from aEE to EoE (83.3 *vs* 66.7%; *P* = 0.6047). As 11 of the 12 cases in the aEE group displayed mucosal edema after extension of linear furrowing, the presence of mucosal edema might be a more reliable predictor of the development of EoE than disease distribution (Fig. 2b). In fact, mucosal edema was observed prior to the emergence of symptoms in two cases in the EoE group (Fig. 2a). Representative endoscopic images of Case 4 were shown in Figure 4. Notably, Case 6 of the EoE group showed stepwise exacerbation, both in endoscopic findings and in symptoms. These results suggest that such change in mucosa over time may offer a clue to the etiology of progression to EoE.

**Table 2:** Baseline characteristics and endoscopic findings for EoE and aEE groups

	EoE (*n* = 6)	aEE (*n* = 12)	Univariate analysis	Multivariate analysis
Chasing period (months)	39.6	43.8	*P* = 0.7330	NA
Period until endoscopically indicated exacerbation (months)	27.0	36.0	*P* = 0.4170	NA
Age	38.2	45.9	*P* = 0.0146	*P* = 0.00643
Gender (male: female)	5:1	10:2	*P* = 1.0000	*P* = 0.5706
Disease distribution (rate of diffuse type [%])	5 (83.3)	8 (66.7)	*P* = 0.6047	*P* = 0.6978
Status of HP infection (rate of receiving HP eradication therapy [%])	1 (16.7)	2 (16.7)	*P* = 1.0000	*P* = 0.8673
History of allergies (%)	2 (33.3)	3 (25)	*P* = 1.0000	*P* = 0.8369

aEE, asymptomatic esophageal eosinophilia; EoE, eosinophilic esophagitis; HP, *Helicobacter Pylori*.

## Discussion

A recent review article mentioned that the prevalence of EoE in Japan has been increasing by up to 0.4%; however, the studies mentioned in the article mainly focused on symptomatic patients, and thus, the precise prevalence of aEE has been unclear.^21^ In this study, we detected 37 cases (0.059%) with aEE in our initial patient registries. This is the second study regarding the prevalence of EE, including aEE in Japan. The first was recently published and reported an estimated EE prevalence of 0.20%.^12^ A much earlier study in Sweden, conducted on the basis of population‐based sampling, indicated an EE prevalence of approximately 0.4%.^19^ In addition, in a study in China, 0.4% of the general population was estimated to have EE.^20^ The reason why our subjects showed a lower prevalence may be due to bias in subject selection. Our subjects were often healthy young adults, ranging in age from 30 to 60 years, and were employees of a limited number of specific companies. The medical histories of EE patients are important in terms of determining the potential for aEE patients to develop EoE, but no study conducted previous to the two Japanese studies included medical history information, and the first Japanese study included information on only one medical history for an aEE case.^12^ They did, however, report that two cases of aEE became PPI‐responsive EE during their respective chasing periods. Of the 37 aEE cases in this study, we were able to consider medical histories in 29 cases, and to our surprise, we confirmed that 18 cases (62.1%) showed exacerbation in endoscopic findings and that 6 cases (20.7%) developed symptomatic EE. Furthermore, only one case (3.4%) was regarded as EoE in the previously mentioned narrow sense of the definition in terms of resistance to PPI therapy. From an epidemiological perspective with respect to our subjects overall, the prevalence of newly developed EoE was estimated to be 0.0096%. As our subjects did not include patients with symptoms, this result does not indicate that the overall prevalence of EoE among Japanese people is lower than that of Western people (estimated to be 0.03–0.52% for a general population).^22^ Reliable epidemiological studies involving a general population will be needed for determining the actual prevalence of aEE and EoE in Japan.

Our findings—that relative youth at first detection and diffuse distribution of linear furrowing appear to be risk factors for disease progression—are consistent with a previous report that younger people, especially those ranging from 30 to 40 years old, were susceptible to EoE.^23^ It is as yet difficult to determine, however, just how EE develops from normal esophageal mucosa, as well as how EoE develops from EE. We have shown three cases of EE newly developed from normal esophageal mucosa, and all three were diagnosed on the basis of linear furrowing in the middle to lower esophagus, without any initial mucosal edema, and two of the three cases showed the appearance of mucosal edema. This reinforces the conclusion that diffuse distribution of linear furrowing is a risk factor for disease progression. In the progressive disease group, all cases experienced an extension of the range of linear furrowing prior to the emergence of mucosal edema. Furthermore, the EoE group, which included PPI‐responsive EE cases, showed common endoscopic findings of mucosal edema. These results suggest a stepwise progression of mucosal change in the development of EoE in which, specifically, eosinophilic infiltration first appears near the esophageal–gastric junction, followed by extension toward the upper esophagus, after which eosinophil infiltration causes mucosal edema, and finally, symptoms related to inflammation and to mechanistic dysfunction of the esophagus become manifest. To confirm this hypothesis, it will be necessary to create a significantly large database of EoE cases that have developed from EE.

This study has several limitations due to the study design. First, patients diagnosed with aEE must be underestimated because this study is based on the retrospective analysis and could not cover all candidates of the general population in Japan and all potential aEE cases because not all of the subjects received a histological examination. Other candidates as potential patients of aEE might be missed in our cohorts. In addition, the possibility of missing other naturally recovered cases is also considered due to the small number of subjects who were assessed successfully. In addition, subjects with complications, including allergic diseases (e.g. bronchial asthma, severe atopic dermatitis etc.), tended to receive screening endoscopy at other more equipped medical institutions. Actually, the prevalence of aEE calculated in this study (0.059%) was much lower than that previously reported, and the frequency of patients with allergic disease was also much lower than expected (27.8% in the progressive disease group and 9.1% in the stable disease group). Second, we were unable to distinguish EE from other eosinophilic disorders, including eosinophilic gastroenteritis, because biopsies from the stomach or duodenum were not performed. However, EoE patients diagnosed in this study did not exhibit any symptoms regarding the gastrointestinal tract except the esophagus. Third, all aEE patients did not undergo esophageal rebiopsies; therefore the judgment of disease progression depended only on the endoscopic findings of extension to a diffuse range of lesions or appearances of mucosal edema. Furthermore, the progressive disease group involved a heterogeneous subpopulation: newly developed aEE cases and cases with endoscopic exacerbation. To overcome these limitations, further reliable prospective studies with larger cohorts should be conducted.

## Supporting information


**Figure S1** Baseline characteristics of the whole cohort. The graph indicates both age distribution and gender ratio in each age‐class.
