# Supplementary material for: Risk of progression to eosinophilic esophagitis in patients with asymptomatic esophageal eosinophilia: A retrospective pilot study
Source: JGH Open. 2019 Oct 11;4(3):422–8. doi: 10.1002/jgh3.12270 (PMC7273718; doi:10.1002/jgh3.12270)
Supplement: Supplementary file 1 — Figure S1 Baseline characteristics of the whole cohort. The graph indicates both age distribution and gender ratio in each age‐class. [file JGH3-4-422-s001.pdf]

Supplementary Figure 1

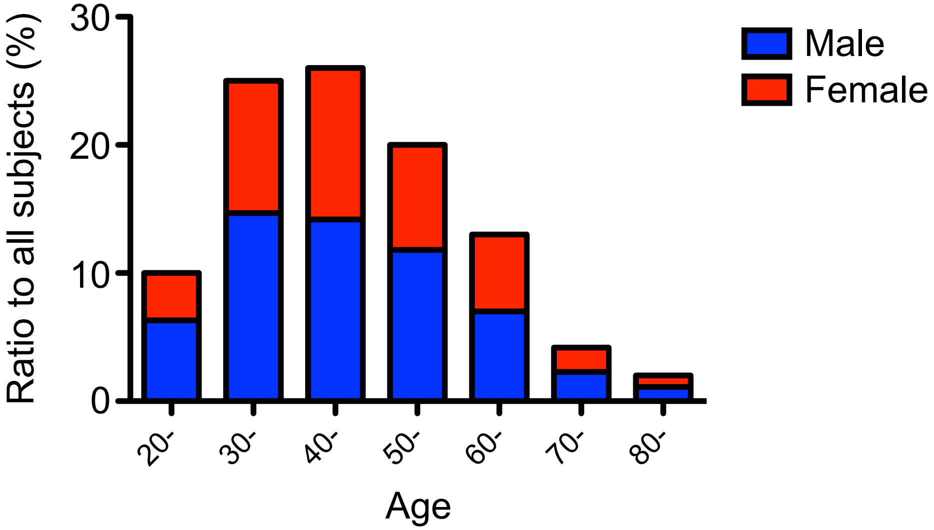

Supplementary Figure legend

Baseline characteristics of the whole cohort. The graph indicates both age distribution and gender ratio in each age-class.
